# The impact of using chickpea flour and dried carp fish powder on pizza quality

**DOI:** 10.1371/journal.pone.0183657

**Published:** 2017-09-05

**Authors:** Hossam S. El-Beltagi, Naglaa A. El-Senousi, Zeinab A. Ali, Azza A. Omran

**Affiliations:** 1 Department of Biochemistry, Faculty of Agriculture, Cairo University, Giza, Egypt; 2 Department of Meat and Fish Technology Research, Food Technology Research Institute, Agricultural Research Center, Giza, Egypt; 3 Department of Crops Technology Research, Food Technology Research Institute, Agricultural Research Center, Giza, Egypt; International Nutrition Inc, UNITED STATES

## Abstract

Pizza being the most popular food worldwide, quality and sensory appeal are important considerations during its modification effort. This study was aimed to evaluate the quality of pizza made using two different sources of proteins, chickpea (*Cicer arietinum*) flour and dried carp fish powder (*Cyprinus carpio*). Analysis indicated nutrients richness specificity of chickpea flour (higher fiber, energy, iron, zinc, linoleic acid and total nonessential amino acids) and dried carp fish powder (higher contents of protein, fats, ash, oleic acid and total essential amino acids) complementing wheat flour to enhance nutritional value of pizza. Total plate count and thiobarbituric acid were increased (*P*<0.05) in dried carp fish powder after 45 days of storage, but no Coliform were detected. Wheat flour was substituted with 5, 7.5 and 10% chickpea flour or dried carp fish powder and chemical, textural, sensory and storage evaluation parameters of in pizza were investigated. Dried carp fish powder increased (*P*<0.05) contents of protein, ash, fats, zinc and protein digestibility of pizza. Chickpea flour increased iron and zinc contents of the pizza. Water activity (*a*_w_) was decreased in fish powder and chickpea pizza. Pizza firmness and gumminess were significantly (*p*<0.05) increased at every level of protein source, but cohesiveness was decreased with 10% chickpea flour. Pizza chewiness was the same (*P*>0.05) across the levels of two protein sources. Springiness was decreased (*P*<0.05) with high level (10%) dried fish powder and low/intermediate level of chickpea flour. Chickpea and dried carp fish incorporation up to 7.50% in pizza at the expense of wheat flour had no effect (*P*>0.05) on all sensorial parameters except for odor values. The results could be useful in utilization of chickpea flour and carp fish powder in designing nutritious pizza for consumers.

## Introduction

Functionality of proteins enables their use in food products in the processing sector [1]. Fish is one of the most important source of animal proteins with the values ranging between 17–20% (on fresh weight basis) [2]. Along with different types of freshwater fishes, Common carp *(Cyprinus carpio*) is the third most widely cultivated and commercially important freshwater fish species in the world [3,4]. According to FAO report- 2013, it contributes about 9% of the world’s total finfish aquaculture production [5]. Although this fish is distinguished by a potential source of good and high protein, fast growth rate, and an easy cultivation way and high feed efficiency ratio, but it has not been fully utilized for its low consumer acceptability and economic value. This is because the fresh form of common carp fish has numerous bones penetrating the flesh, strong fishy smell, softening of the flesh and the limited storage period [3,4]. Accordingly, more innovative processing techniques need to be applied to improve their preservation quality and acceptance by consumers. Dried fish fillets are conventional processed products of fresh-water fish, because they can be stored for a long time and conveniently used [6]. However, processed fish was developed to increase the acceptability and utilization of carp fish [4].

Legumes such as chickpea make a major contribution to the human diet as food sources of protein, carbohydrates, several water-soluble vitamins, and minerals. Raw seeds have limited digestibility, contain certain antinutritional factors and they need preprocessing before they can be eaten [7]. Common domestic processing, including: washing, peeling and soaking are several ways to enhance the availability of healthy nutrients [8,9].

Pizza was introduced in the middle of the 20^th.^ Century. Gradually, it gained huge popularity and now a day's it ranks among the world's most widespread fast foods. It is known for its wide variety and attractive appearance and liked by all aged groups, especially youth [10]. Pizza formulation could consist of the same basic components, but may include an unusually varied choices, such as egg, pineapple, anchovies, banana, coconut, sauerkraut, eggplant, lamb, couscous, fish, and shellfish, chicken tikka masala, and non-traditional spices such as curry and Thai sweet chili [11]. Thus, the previous results recommended that it should be directed towards the utilization of fish protein products like crayfish in food products fortification, especially cereal products up to the concentration of 6–10% depending upon the fortified food product [12]. Also, carp fish protein concentrate had high nutritional value and it was more effective for fortification of some bakery products, whereas, it maximizes the amount of protein content [13]. Thus, the aim of this study was to improve the quality of pizza by substituting wheat flour with different blends of chickpea flour or dried carp fish powder in pizza making and to increase the utilization of chickpea flour and dried carp fish powder.

## Materials and methods

### Raw materials

Freshwater common carp fish (*Cyprinus carpio*) samples were collected from fish markets in Giza city. Chickpea (*Cicer arietinum*) samples and baking ingredients (wheat flour 72% extraction rate, salt, instant active dry yeast, sugar, corn oil and milk) were purchased from local markets in Giza, Egypt. Pepsin and pancreatin were obtained from Sigma–Aldrich Chemical Co. (St. Louis, USA). All other chemicals used were of the analytical reagent grade.

### Preparation of chickpea flour

Chickpea seeds were carefully cleaned and freed from broken seeds and extraneous matter. Then, the sample was soaked in water for 12 h (1:2 w/v), soaked water discarded and chickpea was dried in drying oven at 45±5°C. The dried chickpea was dehulled, milled to fine flour using laboratory mill (IKA-Laboratechnic, Janke and Kunkel Type: MFC, Germany), packed in polyethylene bags and kept at -20°C for pizza preparation and further analyses.

### Preparation of dried carp fish powder

Fresh common carp fishes (average weight 2.50 kg/fish) were carefully washed with running water. Head, scales, fins, gills, viscera, bones and skin were removed. The fishes were washed again with water to remove blood, slime and unnecessary flesh. Fish flesh was cut into pieces and submerged in 1.25% acetic acid solution for 5 min at 25±5°C. After that, these small pieces were boiled in water for 10 min, then dried in an oven at 60±5°C over night till complete drying. Finally, the dried pieces were milled into fine powder and packed in multilayer flexible packages, kept at -20°C for pizza preparation and further analyses.

### Preparation of pizza

Pizza was prepared according to the method described by de Delahaye [14] with minor modification. It contained three different concentrations of chickpea flour or carp fish powder (5, 7.50 and 10%, based on preliminary trials) as partial substitution of bread wheat flour (72% extraction rate). Formula consisted of 100 g flour, 7 ml corn oil, 0.25 g table salt (NaCl), 2 g instant active dry yeast, 1 g sugar and appreciate amount of cow milk with 3.0% fat. Mozzarella cheese, tomato paste and vegetable mixtures were used just for decoration. The prepared pizza was baked at 180–200°C for 15–20 min, packed in polyethylene bags, then subjected to textural profile and sensory analysis. Samples of the prepared product were baked without decoration and dried at 45±5°C overnight, milled and kept at -20°C for further analyses.

### Proximate analysis

Moisture, protein, fat, crude fiber, ash, iron and zinc contents of the dried carp fish powder powder, chickpea flour and pizza samples were measured according to AOAC [15]. Total carbohydrate was calculated by difference. Total calories of samples were calculated according to the formula of James [16].

### Amino acids determination

Amino acid composition of dried carp fish powder and chickpea flour was determined by using an amino acid analyzer (Biochrom 30, based on sodium column, ion-exchange chromatography) according to the method outlined [15]. A known quantity of sample was weighed and digested with 25 ml of 6N HCl at 110°C for 24 h. Then HCl was removed by evaporation; the remaining solid fraction was dissolved with 0.2N sodium citrate buffer (pH 2.2). Amino acid standard containing 17 amino acids was also treated in the similar manner. Amino acids were expressed as g/100 g protein on dry weight basis.

### Fatty acid determination

Dried carp fish powder fats and chickpea oil were extracted with hexane and fatty acids were analyzed by gas chromatography (Trace GC Ultra-Thermo Scientific) and reported in relative area percentages. The methyl esters of fatty acids were prepared according to the method of AOAC [15]. The fatty acid methyl esters were identified using a gas chromatograph equipped with dual flame ionization detector (FID), a capillary column (30m x 0.25mm x 0.25μm) and the carrier gas was nitrogen. The flow rates for hydrogen and air were 50 ml/min and 350 ml/min, respectively. Injector and detector temperatures were 200°C and 220°C, respectively. The fatty acid methyl esters were identified by comparison their retention times with known fatty acid standard mixture. The fatty acid methyl esters were identified by comparing their retention times with known fatty acid standard mixture. Peak areas were automatically computed by an integrator. The fatty acid composition was expressed as percentage of total fatty acids.

### Storage experiment for powdered carp fish

Powdered carp fish were packed in multilayer flexible packages and stored for 45 days. Dried carp fish powder was analyzed for total plate count, coliform and thiobarbituric acid (TBA) at the beginning and at the end of the storage experiment.

### Microbiological analysis

Total plate count (TPC) of dried carp fish powder samples was determined using standard plate count agar [17]. Total coliform bacterial count was determined using Lactose Broth and Brilliant Green Bile, 2% Broth (MPN method) as mentioned by the Food and Drug Administration [18]. The results for the total bacterial count and the total coliform bacteria were expressed as cfu/g and MPN/g, respectively.

### Thiobarbituric acid reactive substances

The thiobarbituric acid (TBA) value was determined colorimetrically by the method of Botsoglou et al. [19]. TBA value was calculated as mg malondialdehyde equivalents/kg dried sample.

### Textural profile analysis of pizza

Texture of whole fresh baked pizza (15x16x1.50cm per one pizza) was determined by universal testing machine (Conetech, B type, Taiwan) provided with software as described by Bourne [20]. An aluminum 25 mm diameter cylindrical probe was used in a Texture Profile Analysis (TPA) double compression test to penetrate perpendicular to 50% depth relative to the sample height, at 1 mm/s speed test (based on pre-test). Firmness (N), gumminess (N) chewiness (N), cohesiveness and springiness were calculated from TPA graphic.

### Water activity (a_w_) of pizza

Baked pizza water activity (*a*_w_) was measured using Rotronic Hygrolab 3 instruments (Model CH-8303, Switzerland) [21]. The measurements were performed in triplicate at ambient temperature (ranged from 21.35 to 22.43°C).

### *In vitro* protein digestibility

The *in vitro* protein digestibility of samples was determined according to the method of Akeson and Stahmann [22]. After enzymatic digestion of samples with pepsin and pancreatin, the protein in the resultant supernatant was estimated using the Kjeldahl method [15]. The percentage of protein digestibility was calculated by the ratio of protein in the supernatant to protein in the sample as the following equation:

$$In\;vitroprotein\;digestibility\;\left(\%\right)=\left(N_{in\;supernatant}\_\;N_{in\;Blank}\right)/N_{in\;sample}x\;100\;N=Nitrogen$$

### Sensory evaluation of pizza

Sensory evaluation of fresh baked pizza was carried out by ten sensory panelists from Food Technology Research Institute according to Larsen et al. [23]. A 9-point hedonic scale was used for determining the sensory evaluation for appearance, pull apart, color, odor, taste, firmness, texture and total score of pizza. The panelists were provided with pizza on a white plate at ambient temperature.

### Statistical analysis

The collected data of raw materials and pizza samples were statistically analyzed in triplicate except for sensory evaluation (n = 10). For the analytical data, mean values and standard deviation are reported. The data obtained were subjected to one-way analysis of variance (ANOVA) at *p*<0.05 followed by Duncan's new multiple range tests to assess differences between group's means. Independent t-test and analysis of variance at *p*<0.05 was used for storage experiments for time zero and 45 days using SPSS version 16.0.

## Results and discussion

### Proximate analysis of raw materials

Chemical composition, iron, zinc and *in vitro* protein digestibility of wheat flour (72% extraction rate), chickpea flour and dried carp fish powder showed in Table 1. The results indicated that chickpea flour had a high content of crude fiber, iron and zinc compared to other raw materials. While, the dried carp fish powder had high contents in protein, fats, ash and total calories compared to other raw materials. Furthermore, wheat flour had the highest values in total carbohydrates and *in vitro* protein digestibility compared to other raw materials. Our results are in agreement with Wu and Mao [24] who found that drying of grass carp fish significantly increase protein content. Also, Livsmedelverket [25] stated that dried fish has 77–87% protein. Wang and Daun [26] found that chickpea has 4.30 to 7.60 mg/100g iron and from 2.80 to 5.60 mg/100g zinc. Regarding protein digestibility, chickpea flour is significant (*p*<0.05) higher in digestible protein percent compared to dried carp fish powder and this is may be due to the effect of soaking and dehulling of chickpea. Chitra et al. [27] reported that chickpea protein digestibility varied from 65.30 to 79.40%.

**Table 1:** Proximate analysis of raw materials (on dry weight basis).

Constitutes	Wheat flour (72% extraction rate)	Chickpea flour	Dried carp fish powder
Fats (%)	1.12^c^±0.13	5.67^b^±0.10	14.39^a^±0.05
Protein (%)	11.31^c^± 0.17	20.00^b^±0.67	79.50^a^±1.42
Crude fiber (%)	0.41^b^±0.02	1.45^a^±0.03	-
Ash (%)	0.61^c^±0.08	2.42^b^±0.08	3.05^a^ ±0.06
*Total carbohydrate (%)	86.96^a^ ±0.33	71.91^b^±0.81	3.07^c^±1.48
**Total calories) kcal/100g(	403.16^c^±0.30	418.67^b^±0.05	459.73^a^ ±0.2
Iron (mg/100g)	3.00^b^±0.08	5.74^a^±0.43	2.98^c^±0.94
Zinc (mg/100g)	1.70^c^±0.02	4.07^a^±0.55	3.24^b^±0.04
***In vitro protein digestibility (%)	80.00^a^±0.12	78.23^b^±0.50	66.00^c^±0.44

*Total carbohydrate was calculated by difference.

**Total calories = Fat x 9 + Protein x 4 + Total carbohydrate x 4.

*** *In vitro* protein digestibility (%) = (*N*_*in supernatant*_ _ *N*_*in Black*_) / *N*_*in sample*_*x* 100 *N* = *Nitrogen* Values are means±SD (n = 3), mean numbers in the same row bearing the different superscript letter are significantly different (*p*<0.05).

### Amino acid contents of chickpea flour and dried carp fish powder

Amino acid contents in chickpea flour and dried carp fish powder are presented in Table 2. Results showed that the dried carp fish powder contained higher amounts of essential amino acids like isoleucine, leucine, lysine, methionine, tyrosine, threonine and valine and higher amounts of nonessential amino acids like glycine and alanine. While, chickpea had the highest content of phenylalanine as an essential amino acid and serine, glutamic, proline, cysteine, argenine and aspartic as nonessential amino acids. The total essential amino acids in chickpea flour and dried carp fish powder were 35.30 and 40.84 g/100g protein, respectively, indicating superiority and high quality nature of protein in dried fish. In addition, total nonessential amino acids in chickpea flour and dried carp fish powder were 52.47 and 50.81 g/100g protein, respectively. The results are close to the findings of Wang and Daun [26] and Jónsson et al. [28] for chickpea and carp fish, respectively.

**Table 2:** Amino and fatty acids content of chickpea flour and dried carp fish powder.

Amino acids (g/100g protein)	Chickpea flour	Dried carp fish powder	Fatty Acid (% of total fatty acids)	Chickpea flour	Dried Carp fish powder
Isoleucine	4.04±0.14	4.50±0.08	Myristic (C14:0)	0.32±0.01	1.26±0.05
Leucine	6.97±0.30	7.64±0.14	Tetrasenoic acid (C14:1)	-	0.10±0.01
Lysine	6.41±0.21	8.67±0.15	Pentadecylic acid (C15:0)	-	0.30±0.01
Methionine	1.31±0.04	2.96±0.05	Pentadecenoic acid (C15:1)	-	0.19±0.01
Phenylalanine	5.86±0.20	4.60±0.08	Palmitic (C16:0)	11.54±0.01	19.09±0.26
Tyrosine	3.33±0.11	3.65±0.06	Palmitoleic (C16:1)	0.34±0.01	9.60±0.30
Threonine	3.23±0.11	4.01±0.07	Margaric acid (C17:0)	-	0.34±0.01
Valine	4.14±0.14	4.81±0.08	Heptadecenoic acid (C17:1)	-	0.44±0.01
Total amounts of essential amino acids	35.30±1.20	40.84±0.73	Stearic acid (C18:0)	4.52±0.02	4.04±0.10
Aspartic	10.56±0.36	9.85±0.18	Oleic acid (C18:1)	27.69±0.05	44.39±0.20
Serine	3.54±0.12	3.16±0.06	Linoleic acid (C18:2)	46.14±0.09	9.52±0.15
Glutamic	13.84±0.47	13.09±0.23	γ-Linolenic acid (C18:3n6)	-	0.25±0.01
Proline	3.89±0.13	3.43±0.03	α-Linolenic acid (C18:3n3)	7.95±0.03	0.90±0.03
Cysteine	2.78±0.09	1.70±0.06	Arachidic acid (C20:0)	0.80±0.02	0.14±0.01
Glycine	3.59±0.12	4.67±0.08	Eicosaenoic acid (C20:1)	0.70±0.01	2.41±0.03
Alanine	3.88±0.13	5.67±0.10	Eicosadienoic acid (C20:2)	-	1.00±0.01
Arginine	7.68±0.26	5.97±0.11	γ- linolenic acid (C20:3n6)	-	0.37±0.01
Histidine	2.73±0.09	3.28±0.06	Arachidonic acid (C20:4)	-	0.86±0.07
Total amounts of nonessential amino acids	52.47±1.78	50.81±0.91	Eicosapentaenoic acid (C20:5)	-	0.44±0.02
			Behenic acid (C22:0)	-	0.10±0.01
			Docosadienoic acid (C22:2)	-	0.13±0.01
			Docosatrienoic acid (C22:3)	-	0.10±0.01
			Docosatetraenoic acid (C22:4)	-	0.24±0.01
			Docosapentaenoic acid (C22:5)	-	0.17±0.01
			Docosahexaenoic acid (C22:6)	-	0.63±0.02
			Lignoceric acid (C24:0)	-	0.46±0.02
			Non identified fatty acid	-	2.53±0.01
			SFA*	17.18±0.11	25.73±0.09
			USFA**	82.82±0.22	71.74±0.16
			USFA/SFA ratio	4.82	2.79

*SFA = Saturated fatty acids.

**USFA = Unsaturated fatty acids.

Values are means±SD (n = 3).

### Fatty acid composition of chickpea flour and dried carp fish powder

The percentage of fatty acids of chickpea flour and dried carp fish oil is showed in (Table 2). The results indicated that oil of dried carp fish powder had high content of fatty acids except for C18:0, C18:2, C18: 3n3 and C20:0. Furthermore, it could be noticed that the main fatty acids in dried carp fish oil were oleic acid (44.39%), while the main fatty acid for chickpea was linoleic acid (46.14%). In addition, chickpea oil had higher unsaturated fatty acid (USFA%) content and USFA/SFA ratio, whereas dried carp fish oil had higher content in saturated fatty acids (SFA). The results are close to Hoseini et al. [29], who found that carp fish contained high amounts of USFA compared to SFA percent (as % of total fatty acids) and oleic acid was the predominant unsaturated fatty acid. Moreover, the SFA ranged from 26.80–28.06%. SFA in carp fish has been reported to be in the range of 27.27–39.90%, while USFA/SFA ratio ranged from 1.50–2.68 [30]. Chickpea had a higher content of oleic acid and linoleic acid compared to other edible pulses and linoleic acid is the dominant fatty acid in chickpea followed by oleic and palmitic acid [31]. The results corroborate with Wang and Daun [26], who found that the fatty acids content in chickpea oil ranged from 8.52–11.0%, 18.44–42.46% and 42.25–65.25% for palmitic, oleic and linoleic acid, respectively.

### Storage parameters of dried carp fish powder

The results of the microbiological analysis of dried fish samples (at time zero and after 45 days of storage) are shown in Table 3. The values of total plate count (TPC) were 1.40x10^2^±1.22 cfu/g at time zero and increased (*p*<0.05) after 45 days to 1.60x10^2^±2.10 cfu/g, respectively. TPC values were ranged from 7.20x10^3^ to 6.70x10^4^cfu/g in different types of dried fish [32]. Meanwhile, the coliforms were not detected in both dried fish samples. Similarly, no presence of *Escherichia coli* was detected in stored fish sausages that were treated with thermal process [33]. The TPC in dried common carp surimi was 1.70 × 10^1^ cfu/g and coliform were not detected and this may be due to the effect of processing of surimi that destroying microorganisms in tissues [34].

**Table 3:** Storage parameters of dried carp fish powder*.

Time	TPC (cfu/g)	TBA (MDA mg/kg)	Total coliform (MPN/g)
Zero day	1.40x10^2^±1.22	0.192±0.01	ND
After 45 days	1.60x10^2^±2.10	0.31±0.02	ND
Statistics			
t-value	-12.435	-3.340	-
F value	1.320	2.462	-
Sig. (2-tailed			
Zero day	0.000	0.029	-
After 45 day	0.002	0.071	-

*TPC = Total plate count, ND = Not detected, TBA = Thiobarbituric acid, MDA = Malondialdehyde. Values are means±SD (n = 3). Independent t-test and analysis of variance was used for the storage parameters at *p*<0.05

Thiobarbituric acid (TBA) test, malondialdehyde (MDA) released, is a helpful indicator of fish quality that it mainly used for evaluation of oil stability and monitoring of deterioration during fish storage [35]. TBA values are increased during storage. In this experiment, there was an increase of 0.12 mg malondialdehyde per kg (*p*<0.05) during 45 days storage of dried carp fish powder (Table 3). Our TBA values are in the same line with those reported by Abou-Zaid and Elbandy [12], who reported values of 0.78 and 0.16 mg malondialdehyde/kg, respectively for crayfish tail flesh powder and crayfish protein concentrate. Latip et al. [35] reported significant increased (*p*<0.05) in TBA values of both white (from 2.05 to 12.97 MDA/kg) and red carp fish muscles (from 6.24 to 22.58 MDA/kg) on the seventh day of freez-storage.

### Chemical composition of pizza

The chemical composition of pizza with different blends are presented in Table 4. Substitution of wheat flour with dried carp fish powder or chickpea flour significantly increased protein contents of pizza samples compared with control pizza. Protein content, was significantly (*p*<0.05) higher in pizza prepared with carp fish than pizza prepared with chickpea. The dried carp fish powder significantly increased (*p*< 0.05) fat content of pizza. Meanwhile, the increase in crude fiber was pronounced more on pizza with chickpea flour. While, substitution of wheat flour with dried carp fish powder or chickpea flour recorded an increase in ash content, total carbohydrate contents was significantly decreased. Chickpea has been reported as a good source of protein and improves the nutritive value of cereal-based diet [36]. It has been reported that the addition of legume to wheat flour baked products improves the essential amino acid balance of such foods [37]. Total calorie content was increased in chickpea and carp fish pizza compared with wheat pizza, owing to more fats in carp fish and chickpea flour. Concerning iron and zinc content, chickpea pizza had a higher content of the both minerals compared with dried carp fish powder and wheat pizza.

**Table 4:** Proximate analysis of pizza with different blends of dried carp fish powder and chickpea flour (on dry weight basis).

Pizza samples	Fats (g/100g)	Ash (g/100g)	Protein (g/100g)	Crude fiber (g/100g)	Total Carbohydrate* (g/100g)	Total calories (kcal/100g)	Iron (mg/100g)	Zinc (mg/100g)
Wheat control	3.62^d^±0.02	0.80^c^ ±0.10	11.40^f^±0.11	0.39^c^ ±0.02	84.20^a^ ±0.13	414.94^c^±0.31	3.07^a^±0.46	1.61^e^±0.01
5% dried carp fish	3.93^bc^ ±0.05	0.89^ab^±0.01	13.35^c^±0.08	0.40^bc^±0.01	81.83^d^±0.13	416.05^b^±0.20	3.10^a^±0.10	1.66^d^±0.02
7.50% dried carp fish	4.12^ab^±0.06	0.90^ab^±0.02	14.30^b^±0.31	0.41^bc^±0.01	80.69^e^ ±0.16	417.01^a^±0.10	3.14^a^ ±0.02	1.72^bc^±0.02
10% dried carp fish	4.31^a^ ±0.03	0.93^a^ ±0.02	15.34^a^±0.18	0.42^bc^±0.01	79.42^f^± 0.22	417.85^a^±0.06	3.20^a^±0.05	1.77^b^±0.11
5% chickpea flour	3.71^cd^±0.28	0.83^bc^±0.03	11.80^e^±0.03	0.44^abc^±0.03	83.67^b^ ±0.32	415.26^bc^±1.36	3.26^a^±0.14	1.70^cd^±0.05
7.50% chickpea flour	3.81^cd^±0.02	0.87^abc^±0.02	12.17^d^±0.04	0.46^ab^±0.05	83.15^c^±0.06	415.57^bc^±0.06	3.32^a^±0.11	1.75^bc^±0.02
10% chickpea flour	3.84^c^±0.08	0.89^ab^±0.01	12.30^d^±0.36	0.49^a^ ±0.06	82.97^c^±0.46	415.62^bc^±0.15	3.45^a^ ±0.04	1.83^a^±0.03

* Total carbohydrate was calculated by difference. *Total calories* = *Fat x* 9 + *Protein x* 4 + *Total carbohydrate x* 4

Values are means±SD (n = 3), mean numbers in the same column bearing the different superscript letter are significantly different (*p*<0.05).

### Texture characteristics

Texture characteristics of pizza prepared from dried carp fish powder or chickpea flour are demonstrated in Table 5. Substitution of wheat flour with dried carp fish powder or chickpea flour in the formulation of pizza resulted change in the pizza textural properties. Pizza firmness, gumminess significantly (*p*<0.05) increased and chewiness was unchanged with increasing percent substitutions of dried carp fish powder or chickpea flour compared with wheat pizza. Our results are in agreement with Gularte et al. [38] who found that the addition of legumes induced an increase in hardness and chewiness of cake. Similarly, Cakmak et al. [39] reported no significant change in the firmness of enriched white bread samples containing chicken meat powder. But, firmness of whole wheat bread was increasing with the increasing level of enrichment and there was a decrease in cohesiveness and springiness values in all chicken pizza samples compared with wheat pizza.

**Table 5:** Texture characteristics of pizza with different blends of dried carp fish powder and chickpea flour.

Pizza samples	Firmness (N)	Cohesiveness	Gumminess (N)	Chewiness (N)	Springiness
Wheat control	1.29^f^±0.02	0.66^a^±0.14	0.86^b^±0.18	0.67^a^±0.17	0.77^a^±0.04
5% dried carp fish	1.64^e^±0.01	0.68^a^ ±0.02	1.12^a^ ±0.03	0.78^a^±0.04	0.70^ab^±0.02
7.50% dried carp fish	1.76^d^±0.03	0.65^ab^±0.05	1.14^a^±0.09	0.76^a^±0.18	0.67^ab^±0.11
10% dried carp fish	1.87^b^±0.02	0.62^ab^±0.02	1.16^a^±0.01	0.70^a^±0.11	0.60^b^±0.08
5% chickpea flour	1.82^c^±0.02	0.61^ab^±0.01	1.11^a^±0.02	0.72^a^±0.05	0.64^b^±0.04
7.50% chickpea flour	1.88^b^±0.01	0.57^ab^±0.03	1.07^a^±0.06	0.70^a^±0.09	0.65^b^±0.05
10% chickpea flour	1.94^a^±0.02	0.54^b^±0.04	1.05^a^± 0.09	0.73^a^±0.07	0.69^ab^±0.01

N = Newton. Values are expressed as means±SD (n = 3). Mean numbers in the same column bearing the different superscript letter are significantly different (*p*<0.05).

### Water activity (a_w_) of pizza

**Fig 1:**
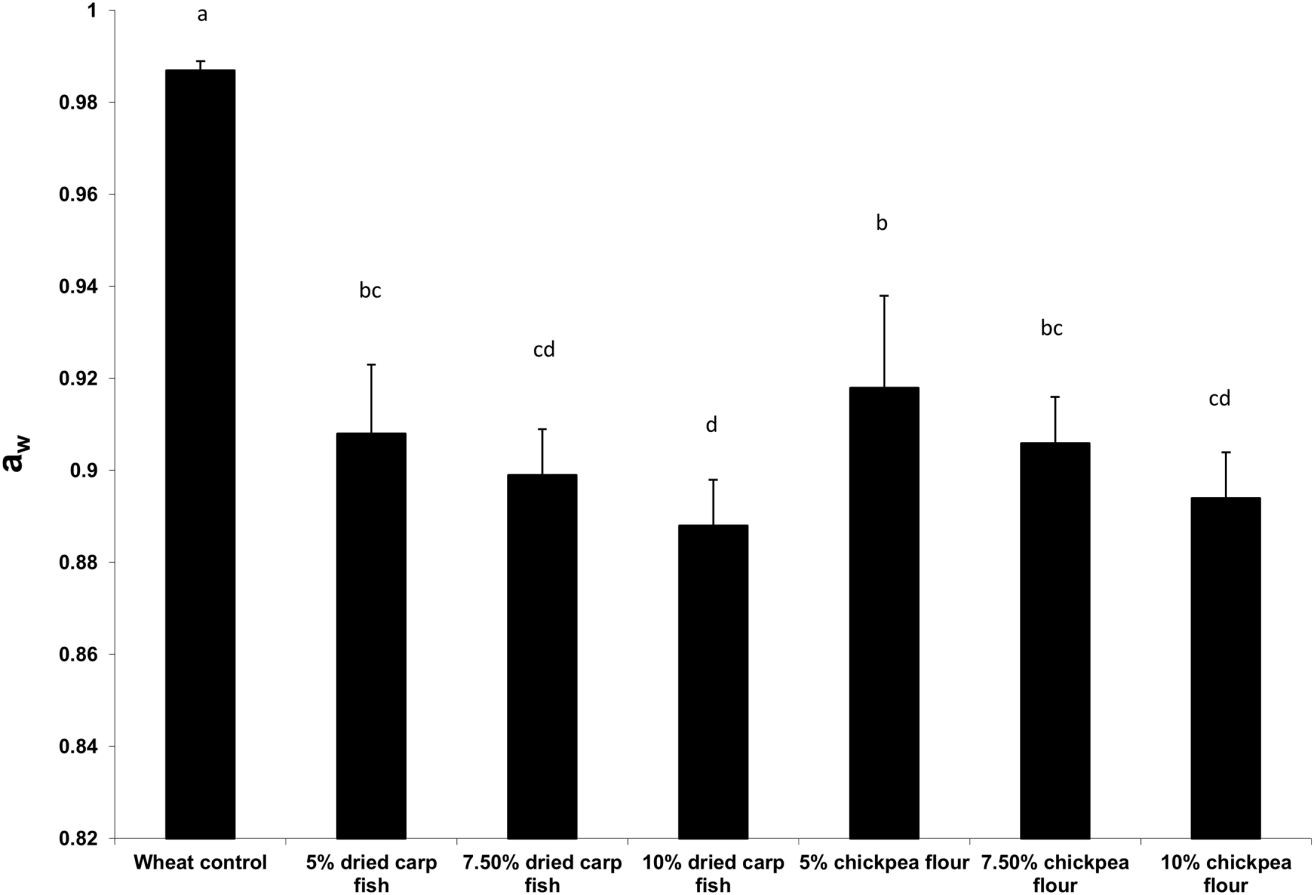
Water activity (a_w_) of pizza with different blends of dried carp fish powder and chickpea flour. Values are means±SD (n = 3), mean represented as bar bearing the different superscript letter are significantly different (*p*<0.05).

Substitution of wheat flour with dried carp fish powder or chickpea flour significantly (*p*<0.05) affected water activity (a_w_) values of pizza samples (Fig 1). While, control pizza (100% wheat flour) recorded the highest a_w_ (0.987), fish pizza exhibited the lowest a_w_ (0.888 to 0.908) and chickpea pizza the intermediate values (0.894 to 0.918). Abbas et al. [40] reported that drying or freezing of the fish can retard the spoilage of fish by reducing the a_w_ and keep the fish in good stage retaining nutritional and organoleptic quality. Reduction of a_w_ increases the shelf life as it reduces the availability of water for the microbial growth [41]. Almost all microbial activity is inhibited below a_w_ = 0.60. Most of the fungi, yeasts and bacteria are inhibited below a_w_ value of 0.70, and 0.80 and 0.90, respectively [42]. Pizza is characterized by specific water activity values, which allow their marketability for a specific period of time. Shelf life is limited by microbial spoilage and staling and the initial a_w_ value of baked pizza was 0.959 [43]. Smith and Simpson [44] reported that pizza being a high moisture bakery product and a_w_ value was 0.99.

### *In vitro* protein digestibility of pizza

**Fig 2:**
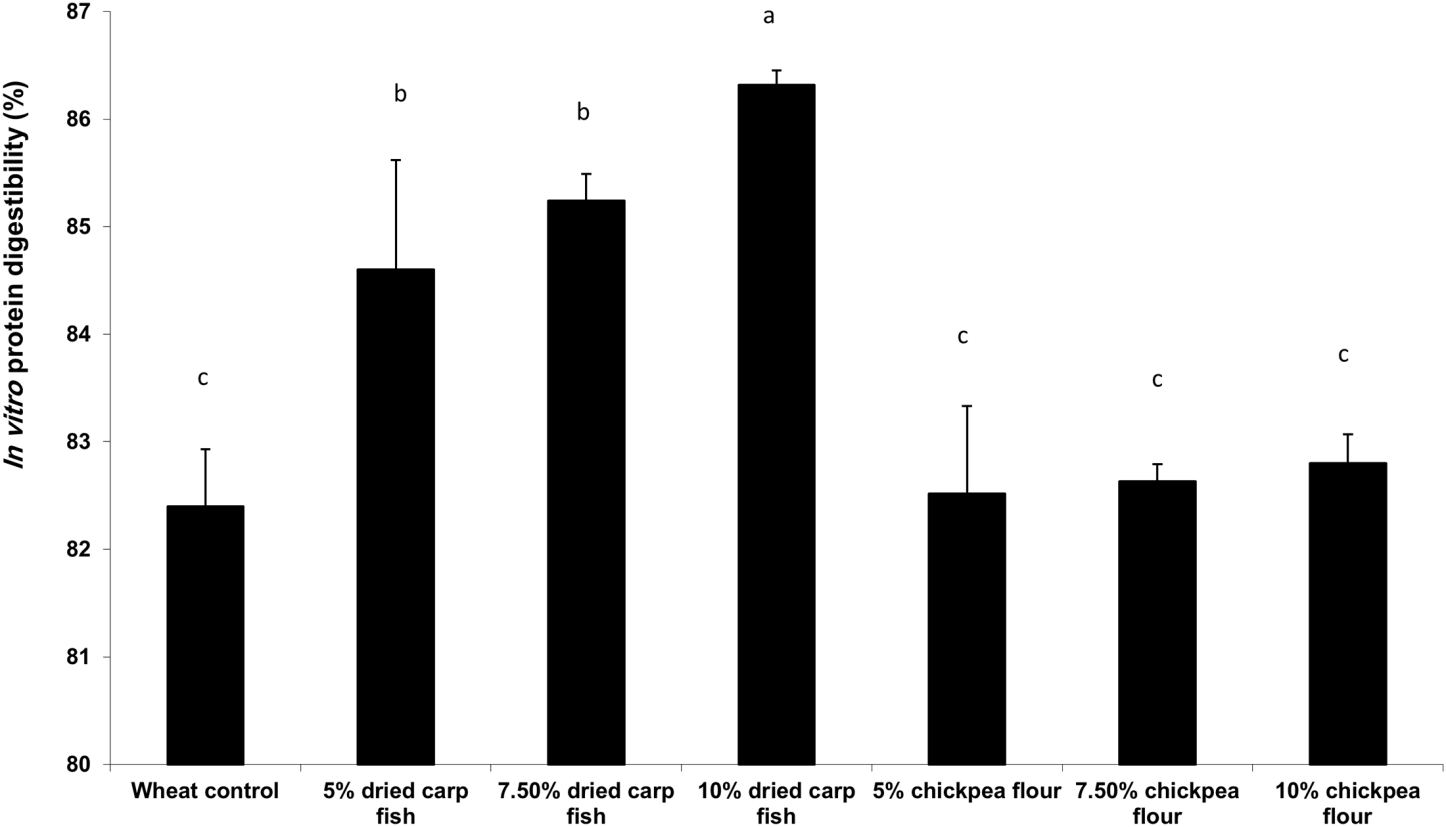
*In vitro* protein digestibility (%) of pizza with different blends of dried carp fish powder and chickpea flour. Values are means±SD (n = 3), mean represented as bar bearing the different superscript letter are significantly different (*p*<0.05).
$$In\;vitroprotein\;digestibility\;\left(\%\right)\;=\;\left(N_{in\;supernatant}\_\;N_{in\;Blank}\right)/N_{in\;sample}x\;100\;N=\;Nitrogen.$$

Substitution of wheat flour with chickpea flour or dried carp fish powder in pizza samples increased the *in vitro* protein digestibility compared with wheat flour pizza (Fig 2). Protein digestibility increased with increasing level of substitution and 10% of dried carp fish powder or chickpea was the highest one and it may be due to that fish muscle is easy to digest and to the effect of dehulling and soaking of chickpea. Our present findings are in accordance with Bilgiçli et al. [45], who reported that protein digestibility is an essential factor when evaluating the protein quality and nutritional status of a food product. Processing of legumes increases the digestibility and enhances the aroma, sensory characteristics and nutritional qualities. This is most likely by destroying protease inhibitors and by denaturing other protein globulins highly resistant to proteases in the native state. Protein digestibility was significantly improved by dehulling peas and faba beans, but dehulling did not affect protein digestibility of chickpeas [46]. The protein in fish protein powder is more concentrated than in the original fish flesh and it is an excellent source of highly digestible amino acids [47].

### Organoleptic characteristics of pizza

One of the limiting factors for consumer acceptability is the organoleptic properties, *e*.*g*. appearance, pull apart, color, odor, taste, firmness, texture and the total score and are performed in Table 6.

**Table 6:** Sensory evaluation of pizza with different blends of dried carp fish powder and chickpea flour.

Pizza samples	Appearance	Pullapart	Color	Odor	Taste	Firmness	Texture	Total score
Wheat control	8.76^a^±0.42	8.55±0.76^a^	8.70^a^ ±0.58	8.68^a^±0.43	8.55^a^±0.47	8.46^a^±0.60	8.50^a^ ±0.50	8.59^a^ ±0.45
5% dried carp fish	8.70^a^ ±0.43	8.46^a^ ±0.97	8.60^a^ ±0.57	8.28^ab^ ±0.48	8.33^ab^ ±0.55	8.19^a^ ±0.83	8.28^ab^± 0.79	8.41^ab^± 0.46
7.50% dried carp fish	8.50^a^ ±0.52	8.28^a^ ±1.02	8.58^a^ ± 0.56	7.92^bc^ ±0.77	8.15^ab^ ±0.63	7.92^a^ ± 0.63	7.83^ab^ ±1.03	8.17^ab^± 0.43
10% dried carp fish	8.46^a^ ±0.48	8.46^a^ ±0.96	8.58^a^ ±0.64	7.70^c^ ±0.75	7.74^b^ ± 0.64	7.83^a^ ±0.79	7.83^ab^ ± 1.12	8.07^b^± 0.52
5% Chickpea flour	8.55^a^ ± 0.48	8.30^a^ ±1.02	8.52^a^ ±0.62	8.55^a^ ± 0.47	8.19^ab^ ±0.51	7.83^a^ ±0.82	7.77^ab^ ±0.98	8.30^ab^ ±0.42
7.50% Chickpea flour	8.40^a^ ± 0.49	8.33^a^ ± 0.77	8.46^a^ ± 0.53	8.46^ab^ ± 0.55	8.01^ab^ ± 0.43	7.92^a^ ± 0.67	7.92^ab^ ± 0.83	8.25^ab^ ± 0.41
10% Chickpea flour	8.51^a^ ± 0.55	8.37^a^ ±1.04	8.55^a^ ±0.47	8.40^ab^ ± 0.53	8.05^ab^ ±0.49	7.83^a^ ±0.39	7.51^b^ ± 0.94	8.24^ab^ ± 0.53

Done using 9 points hedonic structured scale. Values are expressed as means±SD (n = 10). Mean numbers in the same column bearing the different superscript letter are significantly different (*p*<0.05).

Pizza was prepared by substitution of wheat flour (72% extraction rate) by 5, 7.50 and 10% carp fish or chickpea flour. The results in showed that pizza produced from carp fish and chickpea flour had acceptable values of taste comparing with wheat control pizza except for the addition of 10% dried carp fish powder, because of the fishy flavor that naturally present and increase with increasing dried carp percent. Appearance, pull apart, color and firmness were non-significant differences (*p*>0.05) compared with wheat control pizza. Odor values of 7.50 and 10% dried carp fish powder pizza were significantly (*p*<0.05) decreased compared with wheat control pizza due to the increase of fishy odor naturally present with increasing dried carp fish powder percent. Moreover, chickpea and dried carp fish powder values for textural and total scores were similar (*p*>0.05) between different blends compared with wheat control pizza except for the addition of 10% dried carp fish powder or chickpea flour. The primary factors in determining the favorability of pizza are taste and texture [48]. Abdel-Kader [49] mentioned that the addition of chickpea, pigeon pea and bean flours improve the nutritive value, textural and organoleptic properties of wheat bread. In another study, incorporating carp fish protein concentrate up to 3 percent at the expense of wheat flour did not cause any significant (*p*>0.05) deleterious effect on the overall acceptability of produced biscuits [13].

### Conclusion

The results of the present study indicated successful use of chickpea flour and dried carp fish powder and enhanced nutritional value of pizza on chemical, textural and sensory qualities. Substitution of wheat flour with 5, 7.50 and 10% chickpea flour or dried carp fish powder improved the protein content and digestibility of the pizza made. Besides, pizza with dried carp fish powder recorded the lowest a_w_ compared with chickpea, implying possibility to increase shelf life. By increasing the substitution level of chickpea flour and dried carp fish powder, the produced pizza became more firm, gummy and chewy, but less cohesive and springy. The 7.50% level of chickpea flour or dried carp fish powder could be considered to be the best substitution level to retain all sensory characteristics of pizza. Fish powder addition at 10% level impart significant odor to pizza. The results of current study adds value to the science of pizza making. It demonstrates successful utilization of chickpea flour and common carp fish powder to make high quality pizza for the consumers with increased acceptability. Further research should cover different and new processing and drying techniques to increase usage of dried carp fish and chickpea flour as a quality source of protein.

## Supporting information

S1 Fig Water activity (a_w_) of pizza with different blends of dried carp fish powder and chickpea flour. Values are means±SD (n = 3), mean represented as bar bearing the different superscript letter are significantly different (*p*<0.05).
(PDF)

S2 Fig *In vitro* protein digestibility (%) of pizza with different blends of dried carp fish powder and chickpea flour. Values are means±SD (n = 3), mean represented as bar bearing the different superscript letter are significantly different (*p*<0.05).
(PDF)

S1 Tables This is the S1 Tables file. (XLS)
