# Supplementary material for: The impact of using chickpea flour and dried carp fish powder on pizza quality
Source: PLoS One. 2017 Sep 5;12(9):e0183657. doi: 10.1371/journal.pone.0183657 (PMC5584754; doi:10.1371/journal.pone.0183657)

	aw		
**Wheat control**	0.987	0.002	a
**5% dried carp fish**	0.908	0.015	bc
**7.50% dried carp fish**	0.899	0.01	cd
**10% dried carp fish**	0.888	0.01	d
**5% chickp**	0.918	0.02	b
**7.50% chic**	0.906	0.01	bc
**10% chick**	0.894	0.01	cd

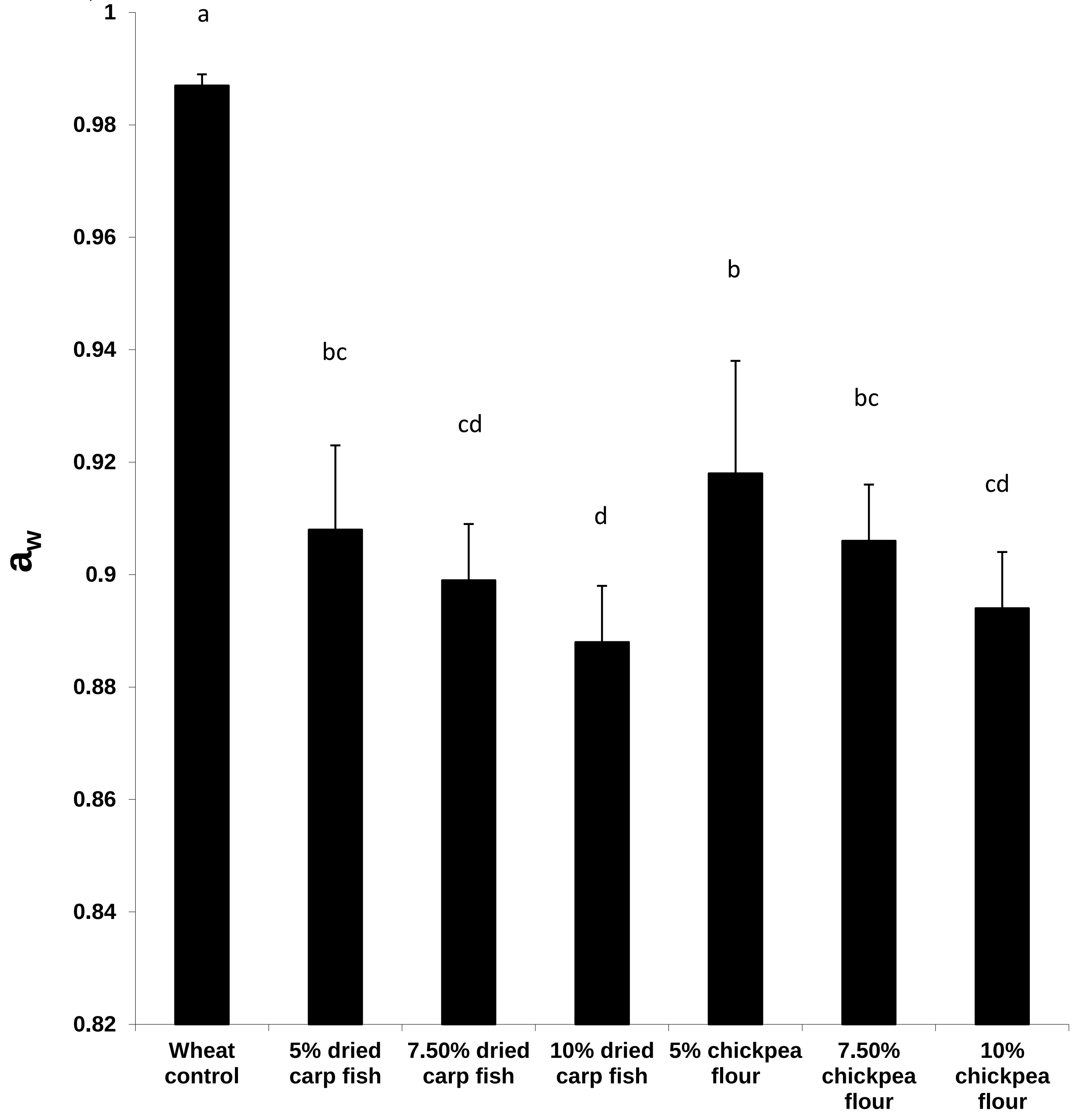

Supplement: S1 Fig — Values are means±SD (n = 3), mean represented as bar bearing the different superscript letter are significantly different (p<0.05). (PDF) [file pone.0183657.s001.pdf]
