# Supplementary material for: The impact of using chickpea flour and dried carp fish powder on pizza quality
Source: PLoS One. 2017 Sep 5;12(9):e0183657. doi: 10.1371/journal.pone.0183657 (PMC5584754; doi:10.1371/journal.pone.0183657)

In vitro protein digestibility (%)

Wheat control	82.4	0.53	c
5% dried carp fish	84.6	1.02	b
7.50% dried carp fish	85.24	0.25	b
10% dried carp fish	86.32	0.13	a
5% chickp	82.52	0.81	c
7.50% chic	82.632	0.16	c
10% chick	82.8	0.27	c

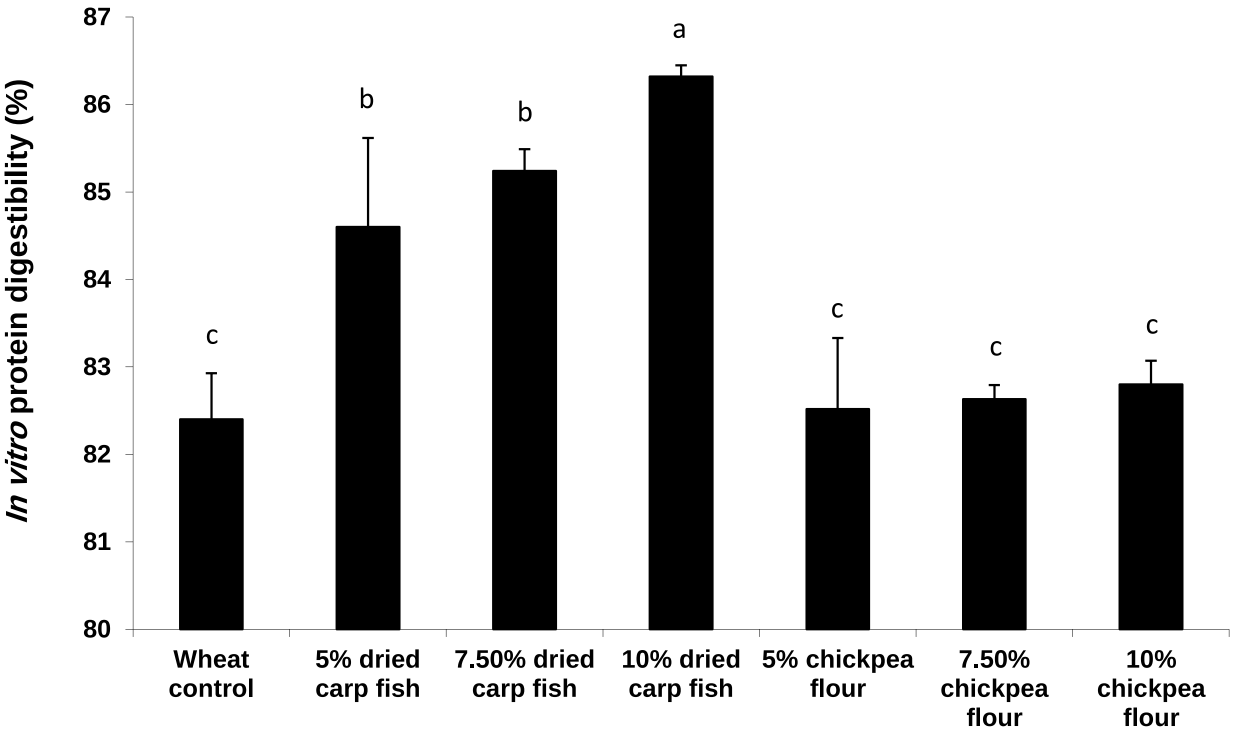

Supplement: S2 Fig — Values are means±SD (n = 3), mean represented as bar bearing the different superscript letter are significantly different (p<0.05). (PDF) [file pone.0183657.s002.pdf]
